# Editorial: Surface EMG and other measurement techniques in rehabilitation research and practice: are new educational programs needed?

**DOI:** 10.3389/fresc.2025.1565879

**Published:** 2025-02-14

**Authors:** Marco Tramontano, Sheng Li, Roberto Merletti

**Affiliations:** ^1^ Department of Biomedical and Neuromotor Sciences, University of Bologna, Bologna, Italy; ^2^ Unit of Occupational Medicine, IRCCS Azienda Ospedaliero-Universitaria di Bologna, Bologna, Italy; ^3^ Department of Physical Medicine and Rehabilitation, McGovern Medical School, University of Texas Health Science Center, Houston, TX, United States; ^4^ LISiN, Department of Electronics and Telecommunications, Politecnico di Torino, Torino, Italy

**Keywords:** physiotherapy, movement sciences, education, sEMG, surface electromyography

**Editorial on the Research Topic**
Surface EMG and other measurement techniques in rehabilitation research and practice: are new educational programs needed?

## 1 Introduction

This Editorial deals with the seven articles submitted to the Frontiers' Research Topic “*Surface EMG and other measurement techniques in rehabilitation research and practice: are new educational programs needed?*” which expands the Research Topic “*Surface Electromyography: Barriers Limiting Widespread use of sEMG in Clinical Assessment and Neurorehabilitation*”.

The education curricula in rehabilitation sciences, particularly the foundational components related to Science, Technology, Engineering, and Mathematics (STEM), vary widely in duration and content among the different healthcare professionals in this field and across countries. Some Universities stress STEM education and offer a Ph.D., some do not. In the European Union the BSc academic curriculum of physiotherapists and occupational therapists (PT and OT) lasts 3 years while in other countries lasts 4–5 years. In the USA the BSc is 3 years but becoming a licenced PT/OT requires 7–8 years. Training in interpretation of bioelectric signals (such as sEMG) is generally neglected. Different aspects of this problem have been addressed in many previous articles on the impact of technology in rehabilitation metrology. See, among many others (1–20), and the Editorials and articles of Frontiers' Research Topics (21–24).

## 2 Discussion of contributions

A review of the most relevant articles and international projects in the field of interfacing physical therapy with measurement technology for quantitative assessment of outcome is presented by Merletti. This author points out how the lack of a PhD degree precludes, in many countries, the academic career of therapists and the training of qualified researchers and teachers in the field, leading to a vicious circle and limiting the academic development of the profession. This leads to the need of new figures with better STEM background that enables them to manage all aspects of rehabilitation metrology. Filling this gap requires time, experience, and expertise that cannot be acquired in a 3-year BSc program. Other reasons, unrelated to educational issues, likely play a role in limiting the scientific and professional developments of the existing figures (25, 26).

Gupta and Aggarwal point out that in many human activities change in behaviour have been introduced by authorities in the field before it was accepted as a useful habit. The use of sEMG-based and other metrologies in rehabilitation implies a significant learning effort and therefore a policy is needed to upgrade teaching. While new academic courses may be suggested by scientists and scientific societies, through tutorials and consensus papers, as done by ISEK, enforcement should come from statutory bodies and councils like NCAHP, World Physiotherapy, NHS and HCPC acting on the Ministries of Education.

These authors also support the strategic role of physiotherapists in creating knowledge and applying measurements and STEM methodology to measure outcome and optimize therapies. These are general foundations of Evidence Bases Practice that should inform a 4-year-long BSc as well as Continuous Medical Education including metrology. Strategies are proposed for reducing the gap between the available knowledge in the field of assessment/monitoring and the clinical and research applications of technologies in rehabilitation, movement, and sport sciences.

The contribution by De La Fuente et al. describes a teaching experience, carried out in Chile and Brazil, based on flipped classwork in the 2nd year of a 5-year BSc in physical therapy. Teachers assigned online materials and articles for homework, prioritizing active learning in laboratory (https://lesley.edu/article/an-introduction-to-flipped-learning). This approach trained the students to work on their own in learning from books and articles, as they would do in their professional life. The activities promoted critical thinking and the application of course content to real-life situations. High levels of participant satisfaction were obtained.

Bertoni et al. report results from an online survey of 93 Italian physiotherapy students in the Master's program for musculoskeletal rehabilitation at the University of Genova, Italy. The survey assessed knowledge, awareness, and perception of sEMG among graduate trainees, working in private practice and clinical/hospital settings. Notably, 96.8% considered sEMG only marginally or moderately important in practice, and the same percentage felt underprepared by their BSc program. These findings, linked to limited exposure, led the authors to suggest that fields blending medical and engineering expertise might benefit from training professionals and teachers specialized in clinical technology or rehabilitation engineering, as is being done in The Netherlands (2)^1^^,^^2^.

The current status of physiotherapy academic degrees in Turkey is described in the work of Uzun and Kahraman who state that the widely neglected STEM-based approach should be integrated into physiotherapy educational programs. In Turkey 56 universities offer a 4-year BSc degree in physiotherapy, 31 offer a 2-year MSc degree and 15 offer a 4-year PhD program. While Sports Science Departments in Turkey promote interdisciplinarity by including specialists in computer programming, physics, electronic and mechanical engineering, the academic staff in BSc, MSc and PhD programs of Physiotherapy and Rehabilitation Departments are required to be physical or occupational therapists or MDs.

A few universities offer a course on “Latest Technologies in Rehabilitation” and only one offers a course on “Technology-Based Assessment in Physiotherapy”. Nevertheless, for decades, physiotherapists have been using state-of-the-art systems and sophisticated instrumentation after receiving only some verbal training from salespersons at the time of sale.

In their work, Gizzi and Felici point out that courses on sEMG, for MDs, Health Sciences, Exercise and Sport are extremely rare and propose a syllabus for a minimal course with two blocks of 35 h of lectures (flipped classes) and 35 h of lab training. Textbooks and online teaching material are recommended.

This syllabus is meant a) to provide a basis for discussion and adaptation to the educational entry level in different countries, b) to outline the topics teachers should be very familiar with, c) to define a basic body of sEMG knowledge common to movement and sport scientists across countries.

Xing-Kai Liu et al. recommend that educational policymakers revise the physical therapy curriculum to include the fundamental principles and the application of sEMG techniques as mandatory content. They also recommend a) that educational institutions should partner with technology companies to incorporate state-of-the-art equipment, providing students with opportunities for hands-on experiences, and b) that educators should actively participate in sEMG technology training and incorporate it into their teaching designs. Through these initiatives, the integration of sEMG technology into physical therapy education should enrich the learning experience and prepare students to the rapidly evolving healthcare landscape, where technology and clinical research are increasingly intertwined.

## 3 Conclusions

The authors of this and the previous collection of papers (21) seem to agree on the problems at hand, though they somewhat differ on the solutions. Proposed solutions range from: a) the introduction of sEMG and other metrologies at the BSc level (hard to do within a 3-year program but possible in longer programs or at the MSc level) to b) the introduction of new figures trained to address/manage current and future technological challenges.

Although the contributions to this collection mostly concern sEMG, the same issues apply to many other technologies and to the broader challenge of metrology in physical therapy, occupational therapy, sport, and movement sciences. Carefully considering solutions adopted in some countries may benefit everyone. A general agreement (perhaps a white paper?) on a clearer definition of professionals and their technological training requirements, including artificial intelligence (16), would be extremely helpful. Moreover, despite the growing importance of interdisciplinary and evidence-based approaches within the rehabilitation field, more efforts in academic programs are needed to address the gap in STEM education.

## Footnotes

^1^https://www.tudelft.nl/onderwijs/opleidingen/bachelors/klinische-technologie/bsc-klinische-technologie/ (Accessed January 10, 2025).

^2^https://www.tudelft.nl/en/education/programmes/masters/tm/msc-technical-medicine/ (Accessed January 10, 2025).
